# Impact of palm olein in infant formulas on stool consistency and frequency: a meta-analysis of randomized clinical trials

**DOI:** 10.1080/16546628.2017.1330104

**Published:** 2017-06-14

**Authors:** John B. Lasekan, Deborah S. Hustead, Marc Masor, Robert Murray

**Affiliations:** ^a^ Scientific & Medical Affairs, Abbott Nutrition, Abbott Laboratories, Columbus, OH, USA; ^b^ M&M Arts and Science, LLP, Durango, CO, USA; ^c^ Departments of Pediatrics and Human Nutrition, The Ohio State University, Columbus, OH, USA

**Keywords:** Meta-analysis, randomized clinical trial, palm olein, infant formula, stool consistency, stool frequency

## Abstract

**Background:** Meta-analysis studies have documented that palm olein (PALM) predominant formulas reduce calcium and fat absorption, and bone mineralization in infants, but none have been documented for stool consistency and frequency.

**Objective:** The study objective was to conduct a meta-analysis of published randomized clinical trials (RCTs) on the effect of PALM-based formulas on stool consistency and frequency in infants.

**Design:** A literature search was conducted in BIOSIS Previews®, Embase®, Embase® Alert, MEDLINE® and Cochrane databases. PALM-based RCTs with available stool outcomes were selected and meta-analyzed. Mean rank stool consistency (MRSC, primary outcome) and stool frequency (secondary outcome) were compared between infants fed PALM-based and PALM-free formulas (NoPALM), using random effects model.

**Results:** Nine out of identified16 studies were meta-analyzed. The mean MRSC (scale of 1 = watery to 5 = hard) in the NoPALM-fed infants was lower (softer stools) compared to the PALM-fed infants (mean difference ‒0.355, 95% Confidence Interval [CI] of ‒0.472 to ‒0.239, *p* < 0.001). Difference for stool frequency was not significant (*p* = 0.613).

**Conclusion:** Meta-analysis of RCTs indicated that NoPALM-fed infants have significantly softer stools but similar stool frequencies versus PALM-fed infants, despite differences in study types and design. Future meta-analysis could benefit from including comparison with human milk-fed infants.

## Introduction

Human milk (HM) is recognized as the gold standard source for infant nutrition. As a result, its composition is often used as a model for the design of infant formula. Although this represents one approach to designing infant formula, a second very different process is to approximate the physiologic outcomes of breast milk on the infant. A combination of both approaches is the ideal; however, the physiologic outcome approach is more desirable compared to the compositional approach.

Fats provide a concentrated source of energy to fuel growth and development in infants. Fats and component fatty acids support cognitive development. They also play a role in the absorption of fat soluble vitamins, calcium, and other essential minerals. The fatty acid composition of HM is unique. The predominant fatty acids in HM are oleic acid (OL, 38% of total fatty acids) and palmitic acid (PA, 21%) [1]. About 70% of the PA in HM is on the sn-2 (or beta) position of the triglyceride molecule. Pancreatic lipase specifically hydrolyzes the fatty acids in the sn-1 and sn-3 positions, leaving the PA as a sn-2 monoglyceride, which is particularly well absorbed [1–4]. To emulate the fatty acid composition of HM, many infant formulas incorporate palm olein (PALM) as their major fat [5–12].

PALM and palm oil (PO) are the two major palm tree-derived oils used for nutritional purposes. PO has a higher melting point than PALM, which is the liquid fraction extracted from PO after crystallization at a controlled temperature. The predominant fatty acid in PO is PA (∼44% of total fatty acid) followed by OL (∼39%). In contrast, PALM is predominantly composed of OL (∼42%) followed by PA (∼39%) [13]. Compared to PO, PALM is more liquid at room temperature and has more homogeneous blend of glycerides; consequently, it blends better with other oils. PALM is used in many infant formulas to mimic HM because it provides closer ratios of OL and PA, which are the first and second major fatty acids in HM and PALM.

However, the triglyceride structure of PALM differs dramatically from HM. About 90% of the PA in PALM is located at the sn-1 (or alpha) and sn-3 (or alpha prime) positions [13–15]. In contrast, PA in HM is predominantly located at the sn-2 position. After hydrolysis by pancreatic lipase, PA in PALM is released as a free fatty acid. This fatty acid binds freely with calcium, forming calcium palmitate, a non-absorbable salt. Consequently, both PA and calcium are not well absorbed from PALM predominant formulas [5,6]. Although PALM-containing infant formulas successfully mimic the PA content of HM, they fail to provide the fat and calcium absorption noted with HM.

Several clinical studies [5–12] have demonstrated the impact of the PALM predominant formulas on one or more of the three major physiological outcomes in infants. These outcomes were (a) reduced calcium and fat absorption or retention [5–7,9,14], (b) reduced bone mineral content or bone mineral density [11–16] and (c) harder stool consistency [7–12], which were observed in infants fed PALM formulas compared to those fed PALM-free formulas. The PALM formulas used in these studies were typically high in PA, which are low in sn-2 PAs (Table 1). In contrast, the formulas containing no PALM (NoPALM) were either low in total PA or were synthetic structural fats (BetaPALM). The BetaPALM were similarly high in PA as PALM predominant formulas but differ by having a higher level of sn-2 PAs [17–20]. The outcome benefits reported for NoPALM and BetaPALM were closer to the benefits reported for HM versus PALM formulas [8,16,17].

**Table 1.** Total palmitic acids (PAs) and relative locations (sn-1 and sn-3 versus sn-2) on the triglycerides of palm olein fat, palm olein-free fat, and synthetic structural fat with high beta PAs.

	Total PAs	sn-1 and sn-3 PAs	sn-2 PAs
PALM	High	High	Low
NoPALM	Low	Low	Low
BetaPALM	High	Low	High

PALM, palm olein predominant fat; NoPALM, fat free of palm olein; BetaPALM, synthetic structural fat with high sn-2 (or beta) PA.

Clinical studies examining the effects of PALM-based formulas differed in several parameters. These studies utilized different study designs (parallel and cross-over); different ages of subjects during infancy; different protein-based formulas (milk protein-based, soy protein-based, intact protein-based, or hydrolyzed protein-based); formulas with or without supplemental docosahexaenoic acid (DHA) and arachidonic acid (ARA) in the fat blend; formulas with or without supplemental prebiotics, galactooligosaccharides (GOS); and different forms of formula (ready-to-feed, powder). Published meta-analysis reports [14,21] of these studies on PALM in formulas have demonstrated a consistent negative effect of PALM on fat and calcium absorption, and bone mineralization.

The currently available meta-analysis reports of clinical data addressing the effects of PALM predominant formulas on fat and calcium absorption or retention and bone mineralization were published by Koo et al. in 2006 [14] and Yu et al. in 2009 [21]. Both studies documented that PALM decreases fat and calcium absorption and bone mineralization, but Koo et al. [14] did not evaluate stool consistency or frequency. Yu et al. [21] reported that infants fed BetaPALM had softer stools compared to infants fed PALM, but reported no data on the comparison with PALM-free formulas. Moreover, other studies [9–11] have been published after the meta-analysis reports by Koo et al. [14] in 2006 and by Yu et al. [21] in 2009. The objective of the current study was to conduct a meta-analysis of up-to-date published randomized clinical trials (RCTs) comparing the effect of PALM-based formulas versus NoPALM-based formulas on stool consistency and frequency in healthy infants. We hypothesize that the NoPALM formulas will produce a softer stool consistency and a higher stool frequency compared to the PALM formula in healthy infants.

## Materials and methods

### Search strategy

The search of the literature was conducted using key terms that included palm olein, palm-olein, infant formula, baby formula, toddler formula, stool, stooling, and human to identify human clinical studies in various databases. The databases included BIOSIS Previews®, Embase®, Embase® Alert, MEDLINE®, and Cochrane database. The search was done on studies available up to July 2016. Identified clinical studies were further screened and reviewed based on study eligibility criteria stated below.

### Study eligibility criteria

There were two study eligibility criteria for the meta-analysis study. The first criterion was that the study selected should be an RCT which compared PALM versus NoPALM formulas (not to be confused with studies on BetaPALM formulas). The second criterion was that the selected study must have stool consistency and frequency outcomes.

### Study selection

Computer search of the databases was performed by a staff of the Abbott Nutrition Library Resource Center (Columbus, OH, USA). Search results were initially screened by the library staff and duplicates were removed. Screening and assessment of study eligibility were initially done by JBL. All other authors subsequently reviewed and agreed on the selection. There were no conflicts in the study screening and selection results between the authors because the eligibility criteria were simple and few and the number of studies was small.

### Data extractions

Data for the parameters of interest were obtained from the published article if available; otherwise, they were obtained from the corresponding unpublished study final reports by the study sponsor (Abbott Nutrition, Columbus, Ohio, USA). Data extraction was performed by author DSH (biostatistician), and then reviewed and verified by author JBL. The primary outcome for assessment in this meta-analysis study was mean rank stool consistency (MRSC).The secondary outcome was stool frequency. For stool frequency (number of stools), all selected studies reported numbers of stools per day.

### Study quality assessment

An uncomplicated assessment of the quality of clinical studies that were selected for the meta-analysis was undertaken. Six quality factors were evaluated in the study. The factors were subject’s inclusion/exclusion criteria, double blind, clinically-labeled study products, study sample power estimation, adverse events, and study completion ratio. Intent-to-treat analysis was not included in the assessed quality factors. This is because several of the studies used two period crossover study design, which had determined *a priori* to include only subjects who had both study treatment periods. The assessment was independently done by two of the authors (JBL and DSH) and then discussed and reconciled where there were differences. The quality factors were rated on a score = 0 if not reported in the published paper or final report and on a score = 1 if reported. The scores of the factors were added and recorded as the overall quality factor. A higher overall quality factor score indicated a higher study quality.

### Statistical analyses

Most of the selected studies for the meta-analysis used a five point scale  for MRSC anchored by 1 = watery and 5 = hard. The only exception was the study by Leite et al. [9] which used a 5 point scale, anchored by 1 = hard and 5 = watery. To match the scale of the other studies, the mean of the Leite et al. study [9] was transformed as follows; new mean = 6 – reported mean. The standard deviation (SD) was unaffected by the transformation. For each variable obtained from the parallel group studies, the mean, SD and sample size for each group were entered into the analyses. For the crossover studies (5–7) the correlation between an outcome for the two study products was not available from either the publications or the sponsor’s unpublished study final reports. Therefore, the raw data were retrieved and the correlations were estimated. For each variable obtained from the crossover studies; the mean, SD, sample size, and estimated correlation were entered into the analysis. Each outcome was examined in a univariate fashion. The mean difference between treatment groups, standard error and associated 95% CI were calculated for each study. It was decided *a priori* that the studies were heterogeneous and that the use of random effects model would be most appropriate. Forest plots were prepared to graphically represent the meta-analysis. Publication bias was investigated by the examination of funnel plots subjectively and also using the Duval and Tweedie’s trim and fill method [22]. The classic fail-safe *N* was determined where applicable. Meta-analyses and investigation of publication bias were performed using the software package, Comprehensive Meta-Analysis, version 2 (Biostat, Inc., Englewood, New Jersey, USA).

## Results

### Study search results

**Figure 1.**
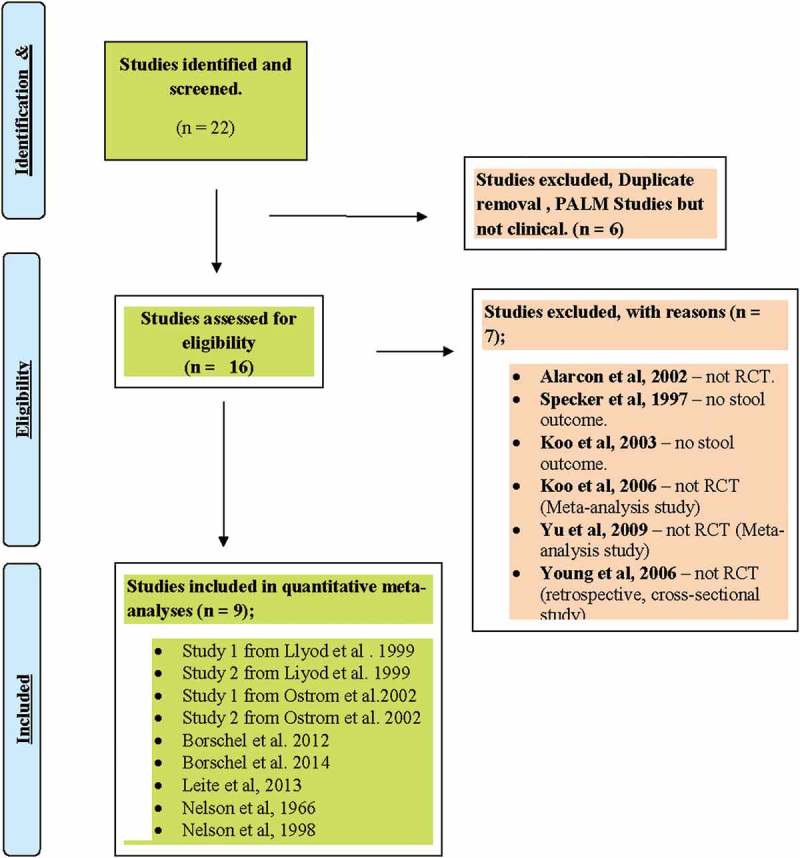
Summary of selection strategy to include studies in the meta-analysis of the effect of NoPALM versus PALM formulas.

The search strategy identified 22 published clinical studies, which compared PALM with NoPALM formulas (Figure 1). After initial screening, six studies were excluded from further screening because of duplications (five studies) and being a human sensory and food science study with no clinical assessment (one study).Nine studies out of the 16 studies remaining met the study eligibility criteria and were included in the meta-analysis (Table 2). Seven studies that did not meet the eligibility criteria were excluded from the meta-analysis (Table 3). One of the studies was the study by Alarcon et al. [12], which was excluded from the meta-analysis because it was not a RCT study, despite having published quantitative stool data in an open label study. The remaining clinical studies [14–16,22–24] excluded from the meta-analysis lacked published stool outcomes or were non-RCT meta-analysis studies. All the included studies clearly indicated that PALM formula was assessed in their studies.

**Table 2.** Characteristics of clinical studies included in the meta-analysis.

					Numbers of subjects/treatment groups (*n*)		
Study	Population	Formula type	Design	Intervention duration	NoPALM	PALM [Control]	Study outcomes
Study 2 from Lloyd et al. 1999 [8]	Healthy term infants	Cow’s milk protein-based Powder	Randomized parallel group.	2 weeks	40	37	Stool consistency & frequency
	12–17 days old.		Double-blinded				GI tolerance
	Canada		Clinically labeled				
Study 1 from Lloyd et al. 1999 [8]	Healthy, term, post-weaning infants	Cow’s milk protein-based Powder	Randomized parallel groups at weaning after Human milk feeding	2 weeks	37	35	Stool consistency & frequency
	4–188 days old.		Double-blinded,				GI tolerance
	United States		Clinically labeled				
Study 1 from Ostrom et al. 2002 [7]	Healthy term infants	Extensively hydrolyzed protein-based	Randomized crossover balance study	2 weeks	11	11	Stool consistency & frequency
	28–87 days old	Liquid	Double-blinded				GI tolerance
	United States		Clinically labeled				Calcium & fat balance
Study 2 from Ostrom et al. 2002 [7]	Healthy term infants	Soy protein-based	Randomized crossover balance study	2 weeks	12	12	Stool consistency & frequency
	40–116 days old	Liquid	Double-blinded				GI tolerance
	United States		Clinically labeled				Calcium & fat balance
Borschel et al. 2012 [11]	Healthy term infants	Partially hydrolyzed whey protein based	Randomized Parallel feeding	12 weeks, used data at 28 days	39	39	Stool consistency & frequency at28 d of age.
	0–8 days old	Powder	Double-blinded				Growth
	United States		Clinically labeled				Bone mineralization
			Evaluable Group analyzed				GI tolerance
Borschel et al. 2014 [10]	Healthy term infants.	Partially hydrolyzed (with added	Randomized Parallel feeding	16 weeks, used data at 28 days	86	77	Stool consistency & frequency at28 d of age
	0–8 days old	prebiotics GOS)	Double-blinded				Growth
	United States	Powder	Clinically labeled Evaluable Group analyzed				GI tolerance
Leite et al. 2013 [9]	Healthy term infants	Cow’s milk protein-based	Randomized crossover balance study (stool data from period 1) only	2 weeks	17	16	Stool consistency & frequency
	68–159 days old	Powder	Double-blinded				GI tolerance
	Brazil						Calcium & fat balance
Nelson et al.1996 [6]	Preterm & term infants.	Cow’s milk protein-based	Randomized crossover balance study	2 weeks	10	10	Stool consistency & frequency
	27–161 days old.	Liquid	Blinded				GI tolerance
	United States		Clinically labeled				Calcium & fat balance
Nelson et al. 1998 [5]	Preterm & term infants (≥ 34 weeks GA).	Cow’s milk protein-based	Randomized crossover balance study	1 week	10	10	Stool consistency & frequency
	22 to 192 days old.	Liquid	Double-blinded				GI tolerance
	United States		Clinically labeled				Calcium & fat balance

GA, gestational age; GI, gastrointestinal; GOS, galactooligosaccharide; PALM, palm olein predominant fat; NoPALM, fat free of palm olein; BetaPALM, synthetic structural fat with high sn-2 (or beta) PA.

**Table 3.** Studies not included in the meta-analysis.

Study	Population	Formula type	Outcomes	Reasons for meta-analysis exclusion
Alarcon et al. 2002 [12].	Term infants	Cow’s milk	Stools	Open feeding study
	17 nations	protein-based	GI tolerance	Non-randomized
				Non-blinded
Specker et al. 1997 [16]	Term infants	Cow’s milk protein-based	Bone mineralization	Non-published stool outcome
	United States		Growth	
Koo et al. 2003 [15]	Term infants	Cow’s milk protein-based	Bone mineralization	Non-published stool outcome
	United States			
Koo et al. 2006 [14]	Term Infants	Various	Calcium absorption & bone mineralization	Meta-analysis
				No stool outcome
Yu et al. 2009 [21]	Infants	Various	Calcium absorption, bone mineralization, & defecation	Meta-analysis
Young et al. 2005 [23]	Children, 4 years of age	Milk protein-based	Bone mineralization	Retrospective study of 4 year olds fed PALM versus NOPALM during infancy
	United States			Non randomized, Non-blinded
				No stool outcome
Innis et al. 1997 [24]	Term infants United States & Canada	Milk protein-based	Growth, visual acuity, and blood lipids	No published stool outcome

The published randomized clinical studies included in the meta-analysis are presented in Table 2. The published study information was supplemented with unpublished stool outcome for two studies [5,6] from the sponsor’s data-on-file report. Out of the nine studies included in the meta-analysis, four studies [8 (studies 1 and 2),10,11] utilized a two treatment parallel study design. Four studies [5,6,7 (Studies 1 and 2)] utilized a two treatment and two period crossover study design. The remaining one study, Leite et al. [9] utilized a two treatment crossover design, but only period 1 stool data from this study was used in the meta-analysis because period 2 stools were for metabolic analyses. Therefore, the study was essentially, a two treatment parallel study.

Data included in the meta-analysis were from a total of 466 infant subjects out of which 423 subjects were in the parallel studies; with 219 in the NoPALM and 204 in the PALM groups. The remaining 43 subjects were in the crossover studies where they received both study products. All subjects in seven of the nine studies were healthy infants with gestational age of 31 to 42 weeks. The two studies by Nelson et al. [5,6] included a low number of preterm infants in their evaluations; however, majority of the subjects in the studies were normal term infants. The age of infants when they were enrolled into the studies varies by study. The age range of the subjects was zero (newborn) to 188 days.

Out of the selected nine studies, eight evaluated cow’s milk protein-based infant formulas. Only one study (study 2 from Ostrom et al. [7]) evaluated a soy protein-based infant formula. Six of the milk protein-based formulas were intact protein-based; two were partially hydrolyzed whey protein based and one was extensively hydrolyzed casein-based. Four out of the nine selected studies evaluated liquid formulations; five assessed powdered formulations. Three recently published studies evaluated formulas containing supplemental DHA and ARA. Only one study evaluated formulas that contained supplemental prebiotics, GOS.

### Publication bias and quality of selected clinical studies

**Figure 2.**
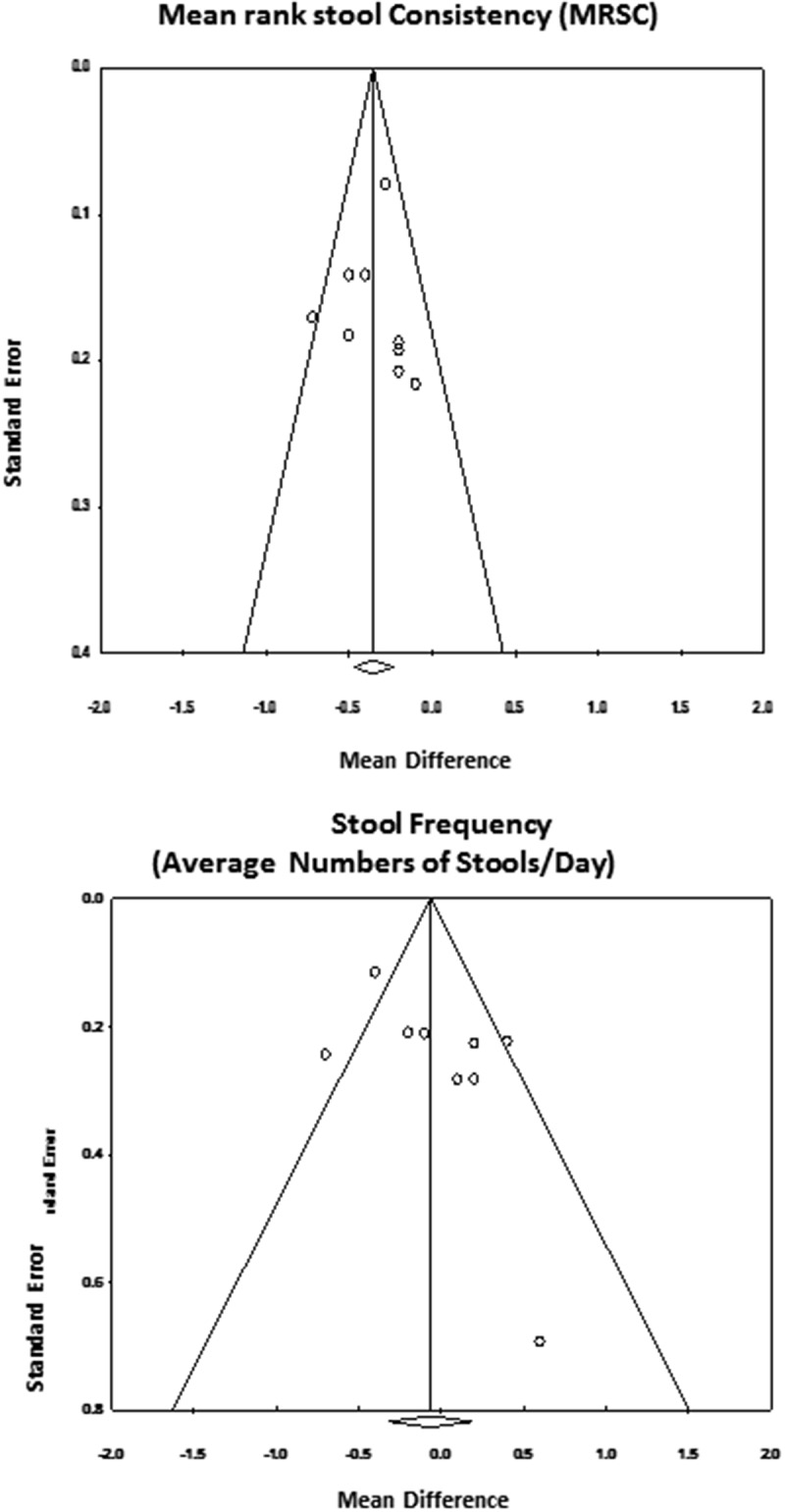
Funnel plots of clinical studies assessing the effects PALM versus NoPALM formulas on mean rank stool consistency and stool frequency.

The results of the assessment of publication bias are presented in Figure 2. For MRSC, the funnel plot appeared to be fairly symmetric. No studies appeared at the very bottom of the plot. Using Duval and Tweedie’s trim and fill approach [22], it was identified there may possibly be one missing study on the left side of the plot. However, the resulting estimated effect size and CI were quite similar to the estimate obtained from the nine studies. The classic fail-safe *N* test indicated that it would take 100 missing studies to be incorporated in order to bring the *p*-value down to less than 0.05. For stool frequency, the funnel plot was fairly symmetric at the top; although, there were no studies in the middle and only one study at the bottom of the plot. Duval and Tweedie’s trim and fill approach fills in one study at the bottom of the plot. As the observed mean difference was not significant, the classic fail-safe *N* test was not assessed for stool consistency.

The quality of the selected studies is presented in Table 4. All the nine studies were RCTs and all studies except Nelson et al. [5] had indications of using a double blind design. Three [5,6,7(study 1)] out of the nine studies did not provide subjects inclusion/exclusion criteria and they tended to be older rather than newer studies. All studies except Leite et al. [9] used clinically labeled products to mask the identity of the study products. However, Leite et al. [9] study had a research staff that measured and distributed the study product without having access to or link to the collected data so as to mask the data and outcome. Adverse events were not reported in four studies [7(studies 1 and 2), 8(studies 1 and 2)]. Study sample power estimations were reported in four studies [8(study 2),9-11] and had adequate power. The overall quality factor score for the nine studies varies between 3 and 6 out of a possible 6. The newest published studies [9–11] had higher scores (5 or 6 scores) compared to the scores (3–5 scores) of the oldest reported studies ([5,6], study 1 by Ostrom et al. [7], study 2 by Ostrom et al. [7], study 1 by Lloyd et al. [8], and study 2 by Lloyd et al. [8]). Overall, the quality of the studies was good. The overall quality scores were 50% and above out of the total possible scores.

**Table 4.** Assessment of the quality of clinical studies included in the meta-analysis *.

Study reference	Study design	Reported inclusion/exclusion criteria	Reported double blind	Reported clinically-labeled products	Reported sample power estimation	Reported adverse events	Reported study completion ratio	Overall quality factor scores
Study 2 from Lloyd et al. 1999 [8]	Randomized Parallel group	1	1	1	1	0	1	5/6
Study 1 from Lloyd et al. 1999 [8]	Randomized Parallel group	1	1	1	0	0	1	4/6
Study 1 from Ostrom et al. 2002 [7]	Randomized Crossover	0	1	1	0	0	1	3/6
Study 2 from Ostrom et al. 2002 [7]	Randomized Crossover	1	1	1	0	0	1	4/6
Borschel et al. 2012 [11]	Randomized Parallel group	1	1	1	1	1	1	6/6
Borschel et al. 2014 [10]	Randomized Parallel group	1	1	1	1	1	1	6/6
Leite et al. 2013 [9]	Randomized Crossover ^1^	1	1	0	1	1	1	5/6
Nelson et al. 1996 [6]	Randomized Crossover	0	0	1	0	1	1	3/6
Nelson et al. 1998 [5]	Randomized Crossover	0	1	1	0	1	1	4/6

*Assessment based on publications and non-published final reports from the study sponsor.

^1^ Stool data were reported only for Period 1 because  period 2 stools were for metabolic analyses; therefore, Leite et al. [9] stool data was reported based on a parallel study design analysis.

### Mean rank stool consistency

**Figure 3.**
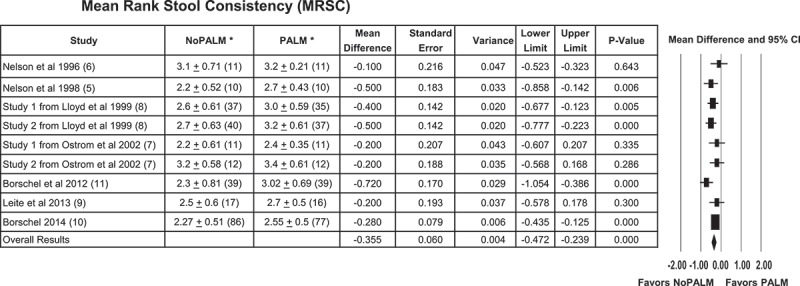
Forest plot results of the effect of PALM versus NoPALM formulas on mean rank stool consistency using random effects model. *Values are means ± SD (*n*). MRSC scales were 1 = watery, 2 = loose/mushy, 3 = soft, 4 = firm, and 5 = hard. PALM, palm olein predominant fat; NoPALM, fat free of palm olein.

The primary outcome was MRSC. The MRSC calculated effect size was the actual differences in the mean, calculated as mean for the NoPALM formula − mean for the PALM formula. In all cases, the mean difference was negative, indicating that the MRSC (scored on a scale of 1 = watery to 5 = hard) for the NoPALM formula was lower (i.e. less hard consistency) than for the PALM formula (Figure 3). This overall calculated difference using all nine studies was significant, *p* < 0.001 with an estimated effect size of −0.355 and associated 95% confidence interval (CI) of −0.472 to −0.239. The observed effect size varies somewhat from study to study. In the meta-analysis study, the observed *Q* was 10.522, a value that is larger than what would be expected if the null hypothesis of common effect sizes is true. However, the p-value was not significant (*p* = 0.230). Consequently, the null hypothesis that all the studies share a common effect size cannot be rejected. The *I*^2^ for the analysis was 23.968. This reflects the proportion of the observed variation that was real, approximately 24%, and therefore the remaining 76% reflects sampling error.

### Stool frequency

**Figure 4.**
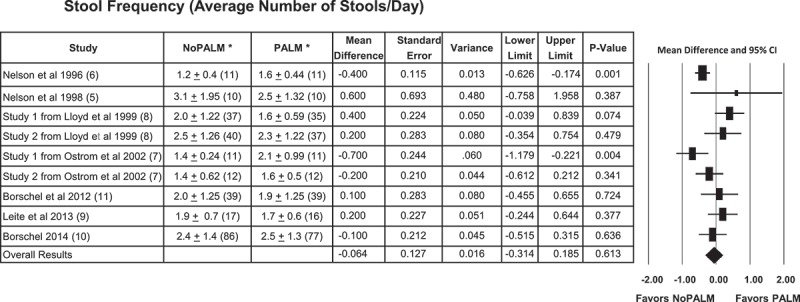
Forest plot results of the effect of PALM versus NoPALM formulas on stool frequency using random effects model. Values are means ± SD (*n*). *PALM, palm olein predominant fat; NoPALM, fat free of palm olein.

The secondary outcome was stool frequency. The stool frequency calculated effect size was the actual differences in the mean number of stools per day, calculated as mean for the NoPALM formula – mean for the PALM formula. For some of the studies the difference was negative, indicating fewer stools per day for the NoPALM formula, and for some of the studies the difference was positive, indicating more stools per day for the NoPALM  formula (Figure 4). Using all nine studies, the overall calculated mean difference was −0.064; non-significant with *p*-value of 0.613 and reflected by an estimated 95% CI of −0.314 to 0.185 which includes zero. The *Q* value was 21.781 with an associated *p*-value of 0.005. Thus, the hypothesis that the studies share a common effect size would be rejected. The *I*^2^ was 63.27.

## Discussion

PO and its fractionated product, PALM, are included in the fat blend of most infant formulas available worldwide in order to mimic the relative amount of PA in HM [5,14,15]. However, the positional distribution of PA on the triglyceride molecules differs from that found on the triglycerides of HM. This difference affects the relative rates of absorption for PA and other fatty acids (2–4), fat, and calcium from PALM formulas [5–7] compared to HM [2–4]. The positional location of PA on the triglycerides of PALM promotes the formation of insoluble calcium fat soaps [18,19], which results in reduced fat and calcium absorption and firmer stool consistency. The calcium fat soap formation is a well-documented reaction, which occurs with long chain saturated fatty acids in the presence of divalent cations, such as calcium. The reaction has been documented in animal studies for over a century [25].

Many studies have demonstrated that fat and calcium were significantly less well absorbed from milk protein-based infant formulas containing PALM as the predominant fat source compared to those absorbed from similar formulas containing NoPALM [1–6,9] or BetaPALM [18–20]. The negative effect of PALM formulas on calcium or fat absorption was also demonstrated when a soy protein-based infant formula and an extensively hydrolyzed casein-based infant formula were evaluated [7]. The effect has also been shown in term [5–7,9] and preterm [5,6] infants. Additionally, studies have reported an increase in the formation of calcium soap-containing hard stools [18,19] in infants fed formulas containing high PA but low sn-2 PA compared to infants fed either similar formulas containing BetaPALM or HM. Harder or firmer stools have also been reported [8(study 1)] in HM-fed infants weaned to a PALM-containing formula as well as those fed such formulas from birth, compared to infants fed NoPALM formula.

Stool consistency is a commonly assessed GI tolerance indicator in infant formula feeding studies [8,26]. It reflects the degree of softness or hardness of stools, which may impact the ease of bowel function, and, possibly, the infant’s mood and behavior. Changes in stool consistency and stool patterns are among the gastro-intestinal (GI) indicators of tolerance to infant formulas in infants. Other indicators may include spit-ups, gassiness, and fussiness [8,26–28]. Tolerance indicators including stool consistency may be impacted by many factors such as differences in the levels and sources of nutrients (ingredients), types of formulations, processing, or combinations of these factors [8,27,28]. Many infant formula studies have demonstrated that inclusion of PALM as the major fat source in formula caused fecal calcium soap excretion and harder stools than those seen in infants fed formulas with NoPALM. The effects of PALM use as the predominant fat source in infant formulas on promoting harder stool consistency have been consistently demonstrated in both milk-protein based [7(study 1), 8(studies 1 and 2),9-12; soy-protein protein-based [7(study 2]; partially hydrolyzed protein-based [10,11] and extensively hydrolyzed protein-based formulas [7(study 1)].

Key studies demonstrating the effect of PALM-based formulas on hard stool consistency include those by Lloyd et al., [8(studies 1 and 2)], which compared the GI tolerance of two milk protein-based powdered infant formulas, one containing palm olein as the major fat (PALM), and the other containing no palm olein (NoPALM). In the first study, [8(study 1)] healthy, full-term infants exclusively HM-fed at the time of enrollment were fed one of the formulas for a two-week feeding period. In the second study [8(study 2)], healthy, full-term infants exclusively fed milk-based formula before enrollment, were fed one of the same formulas for a two-week period. Results of both studies indicated that infants fed the PALM formula experienced significantly firmer and less frequent stools than did those fed the formula with NoPALM. The studies concluded that the stool pattern of softer stools and greater frequency of stooling associated with NoPALM formula were closer to the stool pattern in HM-fed infants. Thus, the studies further suggested that NoPALM formula might ease the transition from breast milk to formula feeding and help ameliorate parents’ perception that constipation or hard stool consistency is associated with iron-fortified formula. The two studies had a good quality factor score(Table 4).

Stool consistency is the only one out of the three major physiological impacts of PALM predominant formulas, which does not have a definitive supportive meta-analysis. The negative impact of PALM on fat and calcium absorption or retention and bone mineralization was nicely demonstrated and published by Koo et al. [14] in a meta-analysis study. The study confirmed the beneficial effects of both NoPALM and BetaPALM. Yu et al. [21] in their meta-analysis similarly evaluated the effects of PALM on both calcium absorption and bone mineralization and defecation. Citing three studies, Yu et al. study [21] concluded that BetaPALM produces softer stools compared to PALM formulas. However, the numbers of studies evaluated were limited and did not include a NoPALM based formula.

Our current meta-analysis study confirms the effect of PALM-based formulas on hard stool consistency, using nine RCT studies that evaluated PALM versus NoPALM formula studies in infants (not to be confused with BetaPALM formula studies). The meta-analysis study provides more pertinent robust data on effects of PALM on stool consistency in infants compared to Yu et al. study [21] by utilizing more studies and more current data. The differences in effect size was observed despite many differences among the nine studies. Variations among the clinical studies include clinical study designs (parallel and cross-over); ages of subjects during infancy; types of protein-based formulas (milk protein-based, soy protein-based, intact protein-based, or hydrolyzed protein-based); formulas with or without supplemental DHA and ARA fat blend; formulas with or without supplemental prebiotics (GOS); and forms of formula (ready-to-drink, powder). Additionally, the studies included in the meta-analysis had above average clinical quality factors.

The results of this meta-analysis study may provide useful information to health care professionals when making formula recommendations. However, the long term medical implications of PALM based formulas are unclear. This study has some limitations. The number of studies used in the study is low. Additionally, we did not find a significant mean difference in stool frequency possibly because of low number of studies. Nonetheless, the results are still reasonable and potentially useful to clinicians and healthcare professionals. Future studies may address these limitations.

In conclusion, the meta-analysis study of available RCTs demonstrated and confirmed that PALM-based formulas produced a harder stool consistency compared to NoPALM formulas in healthy infants despite many differences in clinical study designs, infant subject’s age, formula types, formula composition and ingredients. However, our study did not find a significant effect of PALM on stool frequency. The current meta-analysis study did not include clinical data from infants fed HM or BetaPALM. Future meta-analysis studies could benefit from the inclusion of studies having data on infants fed HM as a comparative gold standard reference.
